# Gene expression profiling supports the hypothesis that human ovarian surface epithelia are multipotent and capable of serving as ovarian cancer initiating cells

**DOI:** 10.1186/1755-8794-2-71

**Published:** 2009-12-29

**Authors:** Nathan J Bowen, L DeEtte Walker, Lilya V Matyunina, Sanjay Logani, Kimberly A Totten, Benedict B Benigno, John F McDonald

**Affiliations:** 1 School of Biology, Georgia Institute of Technology, Atlanta, GA, USA; 2 Petit Institute of Bioengineering and Bioscience, Georgia Institute of Technology, Atlanta, GA, USA; 3 Ovarian Cancer Institute, Atlanta, GA, USA; 4 Department of Pathology and Laboratory Medicine, Emory University School of Medicine, Atlanta, GA, USA

## Abstract

**Background:**

Accumulating evidence suggests that somatic stem cells undergo mutagenic transformation into cancer initiating cells. The serous subtype of ovarian adenocarcinoma in humans has been hypothesized to arise from at least two possible classes of progenitor cells: the ovarian surface epithelia (OSE) and/or an as yet undefined class of progenitor cells residing in the distal end of the fallopian tube.

**Methods:**

Comparative gene expression profiling analyses were carried out on OSE removed from the surface of normal human ovaries and ovarian cancer epithelial cells (CEPI) isolated by laser capture micro-dissection (LCM) from human serous papillary ovarian adenocarcinomas. The results of the gene expression analyses were randomly confirmed in paraffin embedded tissues from ovarian adenocarcinoma of serous subtype and non-neoplastic ovarian tissues using immunohistochemistry. Differentially expressed genes were analyzed using gene ontology, molecular pathway, and gene set enrichment analysis algorithms.

**Results:**

Consistent with multipotent capacity, genes in pathways previously associated with adult stem cell maintenance are highly expressed in ovarian surface epithelia and are not expressed or expressed at very low levels in serous ovarian adenocarcinoma. Among the over 2000 genes that are significantly differentially expressed, a number of pathways and novel pathway interactions are identified that may contribute to ovarian adenocarcinoma development.

**Conclusions:**

Our results are consistent with the hypothesis that human ovarian surface epithelia are multipotent and capable of serving as the origin of ovarian adenocarcinoma. While our findings do not rule out the possibility that ovarian cancers may also arise from other sources, they are *inconsistent *with claims that ovarian surface epithelia cannot serve as the origin of ovarian cancer initiating cells.

## Background

Ovarian cancer is the most lethal of all gynecological cancers [1]. In the United States alone, an estimated 22,000 women will be diagnosed and 15,000 will die from ovarian cancer in 2009 [2]. Despite the obvious clinical significance of the disease, the processes that underlie the onset and progression of ovarian cancer remain among the most poorly understood of all human malignancies.

It has been estimated that up to 90% of ovarian adenocarcinomas are derived from ovarian surface (coelomic) epithelia (OSE) [3,4]. However, in contrast to a *dedifferentiation *origin of adenocarcinomas from more differentiated cells [5], OSE are proposed to become more rather than less differentiated as the malignancy progresses often presenting cellular phenotypes resembling multiple Müllerian or paramesonephric duct derived tissues (*e.g*. endosalpingeal, endometrial and endocervical cells). The lack of morphological similarity between OSE and ovarian adenocarcinomas has led to claims that at least the serous subtype of ovarian adenocarcinoma is not derived from OSE but rather from an as yet to be defined, more differentiated cell residing in the distal end of the fallopian tube [6-9]. Indeed anaplasia and frank carcinoma are often detected in the distal end of the fallopian tube in women harboring germline mutations in either the *BRCA1 *or *BRCA2 *genes that have been estimated to account for more than 80% of *inherited *breast and ovarian cancers [8-10]. However, the inference that some ovarian cancers may arise in the fallopian tube does not detract from compelling evidence that ovarian cancer may arise from OSE [11]. For example, it has been recently demonstrated that murine coelomic OSE display many characteristics of adult stem/progenitor cells, such as *in vivo *label retention and *in vitro *clonogenicity [12]. These findings are consistent with previous studies showing that transformed mouse OSE have the capacity to serve as progenitor cells and differentiate along distinct Müllerian lineages leading to cystic tumors that resemble high-grade serous, endometrioid-like and mucinous-like adenocarcinomas [13]. The fact that cancer stem cells are typically embedded within end stage tumors does not contradict the notion that malignant neoplasms can initiate from the accumulation of mutations in adult stem cell populations leading to their subsequent transformation into cancer *initiating *cells [14]. Indeed, the cancer stem cell hypothesis is the current resurrection of a long suspected origin for cancer [15] and data are rapidly accumulating that support a stem cell origin of many types of cancers [16-18]. The fact that mammalian OSE accumulate high levels of potentially mutagenic 8-oxoguanine modifications following each ovulation is consistent with the proposal that mutant OSE may be the progenitor of ovarian adenocarcinomas [19,20].

To test the hypothesis that human OSE retain properties of relatively uncommitted multipotent progenitor cells until undergoing neoplastic transformation, we conducted gene expression profiling analyses on 12 OSE samples collected *in vivo *and 12 samples of laser capture microdissected cancer epithelia (CEPI) from serous papillary ovarian adenocarcinomas collected from flash frozen tissue. We find that over 2000 genes are significantly differentially expressed between the OSE and CEPI samples. Consistent with a multipotent phenotype, we found that genes previously associated with adult stem cell maintenance are highly expressed in OSE. Pathway analysis implicates key signaling molecules and novel pathway interactions in ovarian cancer development.

## Methods

### Tissue Collection for Microarray

All tissues were collected by the Ovarian Cancer Institute following approved Institutional Review Board protocols from Northside Hospital and Georgia Institute of Technology, Atlanta, GA. Normal ovarian surface epithelial (OSE) cells were collected from ovaries at time of surgery using a Cytobrush^® ^Plus (Medscand), immediately suspended in RNA later (Ambion), and stored at -20°C. Indications for removal of healthy ovaries included other gynecologic pathologies as indicated in Table 1. Tumor tissues were surgically removed and immediately (<1 minute) placed in cryotubes and snap frozen in liquid nitrogen. Following pathological verification, twelve serous papillary cancer samples were embedded in cryomatrix (Shandon). Seven micron frozen sections were cut and attached to uncharged microscope slides for each sample. Immediately after dehydration and staining (HistoGene, LCM Frozen Section Staining Kit, Arcturus), slides were processed in an Autopix Laser Capture Microdissection instrument (Arcturus) and cancer cells captured on CapSure MacroCaps. Approximately 30,000 cancer epithelia were collected from each of the twelve cancer samples.

**Table 1:** Patient Samples Analyzed in this Study

OCI #	AGE AT TIME OF SURGERY	TISSUE FOR MICROARRAY	HISTOPATHOLOGY OF TUMOR OR SURFACE EPITHELIUM	STAGE	GRADE	INDICATION FOR REMOVAL OF HEALTHY OVARIES	MENOPAUSE STATUS
317	59	CEPI	serous adenocarcinoma	Ic	3	N/A	postmenopausal
336	63	CEPI	serous adenocarcinoma	Ic	3	N/A	postmenopausal
367	56	CEPI	serous adenocarcinoma	II	3	N/A	postmenopausal
242	63	CEPI	serous adenocarcinoma	IIb	3	N/A	postmenopausal
183	66	CEPI	serous adenocarcinoma	III	2	N/A	postmenopausal
413	49	CEPI	serous adenocarcinoma	III	3	N/A	postmenopausal
229	58	CEPI	serous adenocarcinoma	IIIc	3	N/A	postmenopausal
369	52	CEPI	serous adenocarcinoma	IIIc	2	N/A	postmenopausal
528	66	CEPI	serous adenocarcinoma	IIIc	3	N/A	postmenopausal
588	71	CEPI	serous adenocarcinoma	IIIc	2/3	N/A	postmenopausal
489	48	CEPI	serous adenocarcinoma	IIIc-IV	3	N/A	perimenopausal
542	61	CEPI	serous adenocarcinoma	IV	3	N/A	postmenopausal
434	41	OSE	WNL	N/A	N/A	atypical complex hyperplasia in polypoid endometrium	perimenopausal
437	54	OSE	WNL	N/A	N/A	cervical adenocarcinoma	postmenopausal
440	50	OSE	WNL	N/A	N/A	uterine myoma	perimenopausal
448	63	OSE	WNL	N/A	N/A	uterine myoma	postmenopausal
452	51	OSE	WNL	N/A	N/A	endometrial adenocarcinoma	perimenopausal
463	48	OSE	WNL	N/A	N/A	endometrial adenocarcinoma	perimenopausal
470	44	OSE	WNL	N/A	N/A	endometrial adenocarcinoma	premenopausal
475	63	OSE	WNL	N/A	N/A	benign simple cyst in right ovary; left ovary brushing used for microarray	postmenopausal
541	41	OSE	WNL	N/A	N/A	adenomyosis uteri and endometriosis	perimenopausal
552	41	OSE	WNL	N/A	N/A	prophylactic TAH-BSO, previous breast and vulval cancer and family history	premenopausal
563	66	OSE	WNL	N/A	N/A	endometrial adenocarcinoma	postmenopausal
567	77	OSE	WNL	N/A	N/A	endocervical adenocarcinoma	postmenopausal

Abbreviations: CEPI - cancer epithelia; OSE - ovarian surface epithelia; WNL - within normal limits; LMP - last menstrual period; HYST - hysterectomy;

TAH-BSO - total abdominal hysterectomy with bilateral salpingo-oopherectomy

### RNA Extraction and Amplification

RNA was extracted from LCM cells on the MacroCaps in 25 μL of extraction buffer and isolated following PicoPure RNA Isolation Kit (Arcturus) protocols. OSE cells were pelleted from RNAlater and RNA was isolated with Trizol (Invitrogen) and purified with the PicoPure RNA Isolation Kit (Arcturus). RNA quality was verified on the Bioanalyzer RNA Pico Chip (Agilent Technologies).

Total RNA from each of the above 24 extractions was amplified using the RiboAmp OA or HS kit (Arcturus) that maintain the original mRNA representation after two amplification rounds, enabling accurate gene expression profiles from ultra small samples. The amplified mRNA was subsequently labeled using the IVT Labeling Kit (Affymetrix), to produce biotin-labeled mRNA suitable for hybridizing to GeneChip Probe Arrays (Affymetrix U133 Plus 2.0).

### Microarray Analysis

Affymetrix.CEL files were processed using the Affymetrix Expression Console (EC) Software Version 5.0. Files were processed using the default MAS5 3' expression workflow. All reported microarray data are described in accordance with MIAME guidelines. The processed and raw data files for the 24 samples used in this study have been deposited in the Gene Expression Omnibus (GEO) http://www.ncbi.nlm.nih.gov/projects/geo/ under the series number GSE14407. Probe sets that were called absent by default MAS5 criteria in all 24 samples were removed before further processing. Probe set results were further evaluated using Spotfire DecisionSite software Probes were considered differentially expressed if they had a fold change value of ≥ 3 and a p-value < .005 (Student's t-test). This resulted in 2915 probe sets differentially expressed between the twelve OSE and twelve CEPI samples. These probe sets were filtered for redundant gene titles (HGU133AV2_V25_affy_annotation file, 3/17/2008) to yield 2320 unique genes being represented by the probe sets. Groups of .CEL files from previous studies were processed in a similar fashion to identify differentially expressed genes. U133 Plus 2.0 probesets were converted to all possible U95 Set probesets using the ID converter application of Babelomics [21] for comparison with the Marquez et al. data [22]. The twelve .CEL files used from the expO study downloaded from GEO were GSM152646.CEL, GSM152724.CEL, GSM102557.CEL, GSM203795.CEL, GSM203744.CEL, GSM117696.CEL, GSM203709.CEL, GSM152581.CEL, GSM179822.CEL, GSM179890.CEL, GSM152659.CEL, and GSM152654.CEL. The significance of the overlap between experiments from different groups was calculated by the hypergeometric distribution statistic calculated in R language, an integrated environment for statistical computing and graphics http://www.r-project.org/.

### Immunohistochemistry

Archival formalin fixed, paraffin embedded tissues from ovarian adenocarcinoma of serous subtype and non-neoplastic ovarian tissues were obtained from the files of Emory University Hospital and Crawford Long Hospital, Atlanta, GA. Emory University's Institutional Review Board (IRB) approved the immunohistochemistry screen of these tissues. The sections (5 microns) were deparaffinized and rehydrated. Antigen retrieval was in citrate buffer (pH 6) using an electric pressure cooker for 5 min at 120°C with cooling for 10 min before immunostaining. All tissues were exposed to 3% hydrogen peroxide for 5 min, primary antibodies for 30 min, DAKO ENVISION system (DAKO Corp) HRP labeled polymer conjugated with secondary antibody for 30 min, diaminobenzidine as chromogen for 5 min and DAKO automated (DAKO AUTOSTAINER) hematoxylin and counterstain for 15 min. Primary antibodies used in this study were anti-ALDH1A2 (Dr Peter McCaffery, University of Aberdeen, UK.), LHX9 (Abcam), and SFRP1 (GenWay). All incubations were performed at room temperature. Between incubations, sections were washed with tris-buffered saline (TBS) buffer. Cover slipping was performed using the Tissue-Tek SCA (Sakura Finetek USA, Inc.) automatic cover slipper. Slides were scored by a board certified pathologist (S.L.). Slides were photographed with an Olympus C5050 digital camera attached to the optical port of an Olympus BX60 compound microscope.

## Results

### Over two thousand genes are differentially expressed between OSE and CEPI

**Figure 1:**
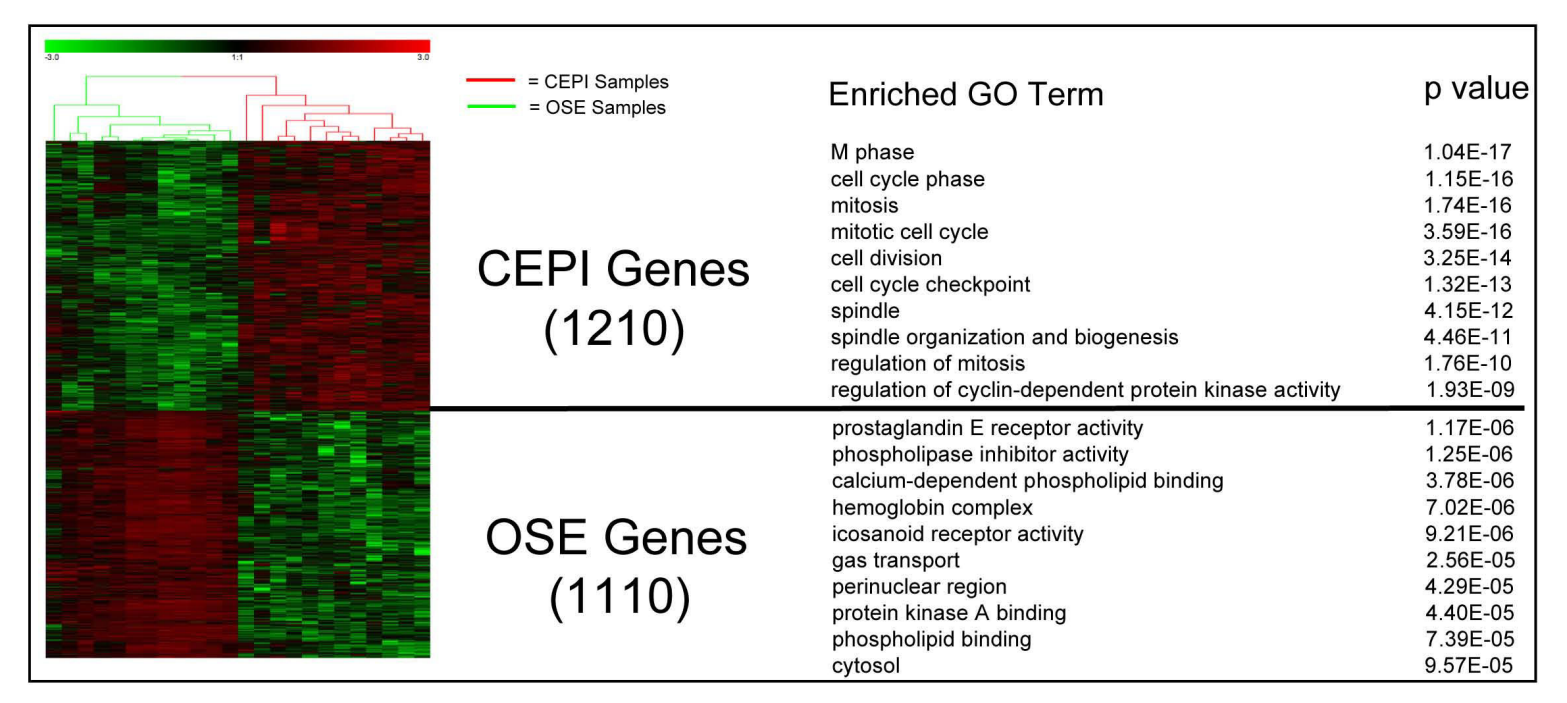
**Hierarchical clustering and gene ontology (GO) enrichment of 2320 genes differentially expressed between ovarian surface epithelial cells and ovarian cancer epithelial tissue**. The heat map (left) was generated by Z-score normalization of log2 expression values from Affymetrix HGU133 Plus 2.0 3. Displayed are the relative expression levels of genes (rows) differentially expressed (red = relatively over-expressed; green = relatively under-expressed) in 12 ovarian surface epithelial brushings and 12 laser capture microdissected malignant epithelia samples (columns). Unique, enriched GO terms are listed for each set of differentially expressed genes and their statistical significance by false discovery rate (FDR) corrected, hypergeometric distribution p-values. Genes overexpressed in CEPI are labeled as CEPI Genes overexpressed in OSE are labeled OSE Genes.

We generated 24 individual gene expression profiles (Affymetrix Human Genome U133 Plus 2.0 Arrays) from 12 OSE brushings and 12 CEPI samples isolated by laser capture microdissection. Relevant histopathologies of the 24 samples are listed in Table 1. A supervised (>3-fold change, t-test p < .005) differential expression analysis between the OSE and CEPI samples, yielded 2915 differentially expressed probe sets (see Additional file 1). After manually removing probe sets corresponding to the same Affymetrix gene title, 2320 differentially expressed genes remained (see Additional file 2). Of these, 1210 genes are highly expressed in CEPI relative to OSE and 1110 are highly expressed in OSE relative to CEPI. A hierarchical clustering of the data resulted in a distinct separation between the OSE and CEPI samples (Figure 1).

### Comparison of gene expression profiles with prior studies

To assess the correspondence between our data and other independently gathered ovarian cancer microarray data sets, we compared differentially expressed probes (DEPs) for significant overlaps using the Bonferonni corrected hypergeometric distribution probability. We first compared our DEPs to those generated after performing an identical differential expression analysis (>3-fold change, t-test p < .005) on five normal ovarian surface epithelial brushings and eleven flash frozen bulk tissue serous ovarian cancers that were previously characterized with the Affymetrix Human Genome U95 Set of 3' Expression Arrays [22]. Our analysis of these data resulted in 1000 DEPs. In order to relate our data to that of this earlier study, all of our DEPs from the U133 Plus 2.0 Arrays were converted to all possible corresponding probe sets from the U95 Set, resulting in 3920 probe sets for comparison. When compared to each other, we detected a statistically significant overlap (392 probe sets, Bonferonni corrected p = 1.09E-94).

We also compared our DEPs to findings from a differential expression analysis of our OSE to twelve serous papillary ovarian cancer bulk tissue microarrays produced independently by the International Genomics Consortium http://www.intgen.org/ Expression Project For Oncology (exp *O*, http://expo.intgen.org/geo/home.do) using the Affymetrix Human Genome U133 Plus 2.0 Arrays. Comparison of our OSE versus the exp *O *bulk tissue data resulted in 9942 DEPs. When compared to our 2915 DEPs, a statistically significant overlap (1498 DEPs, Bonferonni corrected p = 1.19E-252) again was detected.

The DEPs from each of these analyses are presented in Additional file 3. These DEP comparisons provide evidence for significant concordance between our data set and those of previous studies. The differences that remain may be attributable to contaminating stromal, immunological and/or vascular cells contained in bulk tumor samples used in the other studies.

### Genes differentially expressed between OSE and CEPI are involved in canonical cell cycle and signaling pathways

In order to provide a global view of the biological processes associated with genes differentially expressed between OSE and CEPI, we searched for enrichment of functional annotations within the Gene Ontology (GO) database using the *Genomica *software package [23]. To provide sample specific meaning to the differentially expressed genes, we divided our results into two sets: 1) genes that were on average significantly up-regulated (positive fold change values) in CEPI relative to OSE and, 2) genes that were on average significantly down-regulated in CEPI (negative fold change values) relative to OSE. The results indicate that 258 GO terms are unique or significantly enriched (p ≤ 0.05) for genes up-regulated in CEPI (Figure 1 and Additional file 4). Of these, the most significant category (p = 1.17E-06) was "M phase" (i.e., genes involved in mitosis and cytokinesis). Not surprisingly, the top 19 GO terms for genes up-regulated in CEPI were directly related to cell division. In contrast, genes up-regulated in OSE were uniquely enriched for 202 GO terms typically associated with non-dividing or quiescent cells (Figure 1 and Additional file 4). Differences in the expression pattern of signaling pathway ligands, receptors, and downstream transcription factors were used to establish the status of OSE and CEPI cells in key canonical signaling pathways.

#### The cell cycle pathway

**Figure 2:**
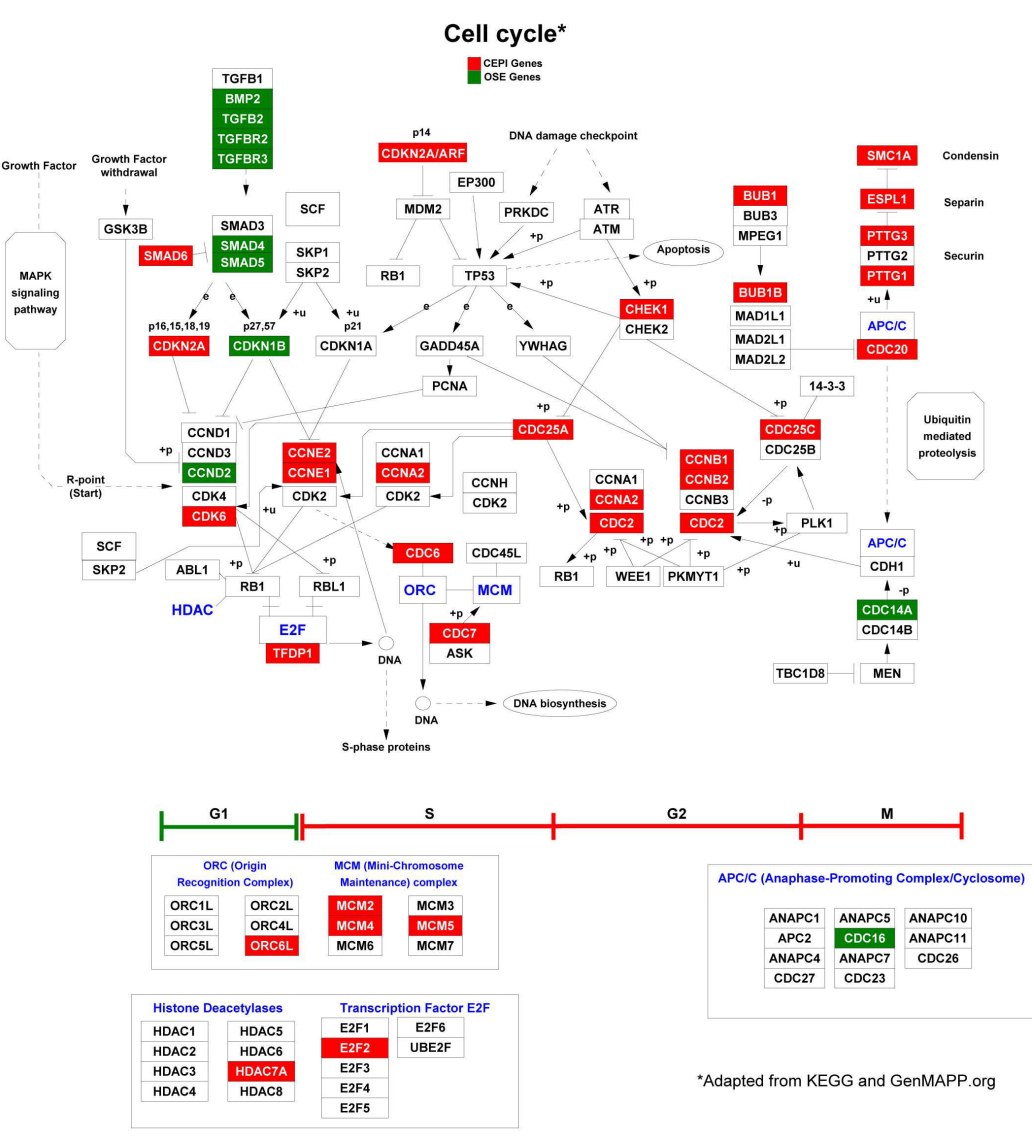
**Cell cycle pathway gene expression**. Shown is a GenMAPP http://www.genmapp.org rendering of a modified KEGG (Kyoto Encyclopedia of Genes and Genomes, http://www.genome.jp/kegg/) schematic of cell cycle pathway genes. Genes significantly overexpressed in CEPI relative to OSE are colored red. The execution of the cell cycle is depicted from left to right and individual phases identified below by I- beam brackets. Genes involved in maintaining G1 are generally under-expressed in CEPI while genes involved in G1 to S progression, G2, and M are over-expressed.

Figure 2 overlays differences in gene expression between OSE and CEPI on the cell cycle pathway. Genes known to be instrumental in maintaining cells in the G0/G1 phases of the cell cycle are highly expressed in OSE. For example, representatives of the transforming growth factor beta (TGFB) signaling pathway, as well as, cyclin-dependent kinase inhibitor 1B (*CDKN1B*) are highly expressed in OSE. In contrast, genes known to be involved in the transition from G1 to the S phase of the cell cycle are highly expressed in CEPI. Examples are the cyclins E1, E2, B2 and A2 (*CCNE1*, *CCNE2*, *CCNB2 *and *CCNA2*), as well as, members of the Origin Recognition (*ORC6L*) and Mini-Chromosome Maintenance (*MCM2*, *MCM4 *and *MCM5*) complexes. The results are consistent with the hypothesis that OSE are arrested in the G0/G1 phase of the cell cycle while CEPI are actively replicating.

**Figure 3:**
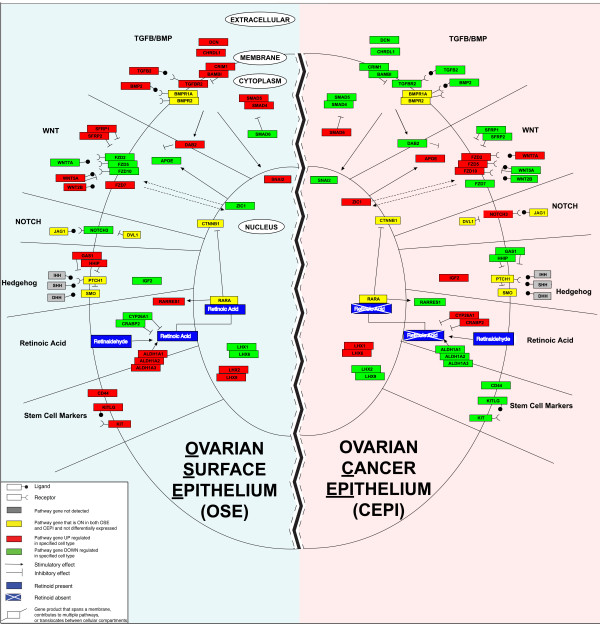
**Pathway deregulation in ovarian cancer**. Individual signaling pathways hypothesized to be deregulated in the oncogenic transformation of ovarian surface epithelia (OSE) into ovarian cancer epithelia (CEPI). An individual OSE is represented on the left with individual pathways (as discussed in the text) labeled adjacent to their respective section of each cell. An individual CEPI with the same signaling pathways is represented on the right as a mirror image of the OSE cell. The legend describes the colored boxes and lines used to represent expression differences and potential interactions among genes. The juxtapositional placement of the two cell halves is meant to emphasize the dichotomous state of signaling between the OSE and CEPI as revealed by our gene expression microarrays.

With the aid of *Pathway Express *[24], we identified from our DEPs specific upstream signaling pathways contributing to the inactive (OSE) and active (CEPI) states of the cell cycle in ovarian cancer development. Figure 3 illustrates the differential expression status of components of each signaling pathway interpreted from both an OSE and CEPI frame of reference. Heatmaps depicting both the log2 expression levels and Z-score normalization of these individual genes are presented in Additional files 5 and 6.

#### The TGFB/BMP Pathway

The TGFB/BMP pathway is known to be an important growth inhibitor of epithelial cells [25]. The results presented in Figure 2 show that the expression of genes mediating TGFB/BMP induced growth inhibition in OSE is significantly down-regulated in CEPI. For example, expression of the transforming growth factor, beta 2 (*TGFB2*), the TGFB2 receptors II and III (*TGFBR2*, *TGFBR3*), disabled homolog 2 mitogen-responsive phosphoprotein (*Drosophila*) (*DAB2*), bone morphogenic protein 2 (*BMP2*), as well as, the BMP receptors *BMPR1A*, and *BMPR2 *are all significantly reduced in CEPI while *SMAD6 *(mothers against decapentaplegic homolog 6 (*Drosophila*)), an inhibitory SMAD [26], is highly expressed in CEPI (Figure 3). Snail 2 (*SNAI2*) [27-29] is expressed at high levels in OSE (Figure 3) consistent with the absence of CDH1 expression in OSE [4]. Extracellular modifiers of TGFB/BMP signaling, decorin (*DCN*)[30], and chordin-like 1 (*CHRDL1*) are also expressed at high levels in OSE [31].

#### The WNT Pathway

The WNT (wingless-type MMTV integration site family members) signaling pathway has been implicated in a variety of normal and disrupted developmental processes such as stem cell maintenance [32], embryonic patterning and cancer [33,34]. The secreted ligands of the WNT family are known to stimulate cellular proliferation through interactions with their cognate frizzled receptors (FZDs) but are also found to inhibit cellular proliferation in cell-dependent contexts [35]. For example, the inhibitors of differentiation, WNT2B and WNT5A [35,36], are expressed at significantly higher levels in OSE (Figure 3). In contrast, WNT7A, an inducer of cellular replication in the female reproductive tract [37], is highly expressed in CEPI. The WNT receptor FZD7 that has been shown to be expressed in embryonic stem cells (ES) and to play a role in self-renewal capacity of ES cells [38], is highly expressed in OSE and significantly down-regulated in CEPI. The high expression of known antagonists of WNT signaling including *WNT5A*, *DAB2*, [39] secreted frizzled related protein 1, (*SFRP1) *and secreted frizzled related protein 2 (*SFRP2) *in OSE indicates that major components of WNT signaling are attenuated on the surface of the ovary. The high expression of WNT7A and several FZD receptors in CEPI alternatively suggests that various components of WNT signaling are activated in CEPI.

Among the most highly expressed genes in CEPI is Zic family member 1(*ZIC1*), an activator of WNT signaling [40]. ZIC encoding genes have been previously implicated in cancer development [41] and ZIC1 is known to transcriptionally trans-activate apolipoprotein E (*APOE*) [42] - a gene previously implicated in the proliferation and survival of ovarian cancer cells [43]. Over-expression of *ZIC1 *in CEPI is significantly correlated with *APOE *expression (*Pearson's r *= +0.65).

#### The NOTCH Pathway

The complex interactions that exist between signaling pathways are readily evident in our data. For example, the WNT pathway is known to have recurrent and consistent interactions with the NOTCH signaling pathway [44]. The NOTCH signaling pathway plays an important role in cell-to-cell communications that regulate multiple cell differentiation processes during embryonic and adult life [45]. NOTCH3 has been identified as being frequently over-expressed in ovarian cancer cells and is thought to form a juxtacrine signaling loop with the ligand jagged1 (*JAG1*) produced by mesothelial cells of the intraperitoneal cavity [46,47]. NOTCH3 over-expression also has been shown to play a major role in the proliferation of ERBB2-negative breast cancer cells [48]. Our results demonstrate that *NOTCH3 *is highly expressed in CEPI and expressed at significantly lower levels in OSE.

#### The Hedgehog Pathway

Activation of the Hedgehog (Hh) signaling pathway has previously been implicated in multiple cancers [33,34]. Consistent with activation of the Hh signaling pathway in CEPI, our results show that two known antagonists of Hh signaling, hedgehog interacting protein (HHIP) and growth arrest-specific 1 (GAS1) [49,50] are expressed at low levels in CEPI. In addition, genes previously shown to be inversely regulated following Hh pathway activation in pluripotent mesenchymal cells (e.g., insulin-like growth factor 2 (*IGF2*) is up-regulated while *SFRP1 *and *SFRP2 *are down-regulated) [51], are likewise inversely regulated in CEPI.

#### The Retinoid Pathway

Retinoids are vitamin A-derived morphogens that can directly modulate WNT signaling during normal development [52]. The gene with the largest fold decrease (-256×) in CEPI is aldehyde dehydrogenase 1 family, member A2 (*ALDH1A2*). This gene encodes an enzyme responsible for the conversion of retinaldehyde to retinoic acid - a known marker of lineage specific stem cells [53]. The low levels of ALDH1A2 in CEPI imply that a deficit of retinoic acid in these cells may be contributing to ovarian cancer by attenuation of the WNT signaling pathway. Other genes known to modulate the cellular activity of retinoic acid are also differentially expressed between OSE and CEPI (e.g., cytosolic cellular retinoic acid binding proteins (*CRABPs*) [54,55] and cytochrome P450s (*CYP26A1*) [56,57]). The fact that genes involved in the synthesis/activation of retinoic acid are not expressed in CEPI while genes involved in the degradation and/or inhibition of retinoic acid signaling are expressed in CEPI strongly implicates alteration of the retinoid signaling pathway in ovarian cancer development.

#### Immunohistochemistry validation of differentially expressed genes

**Figure 4:**
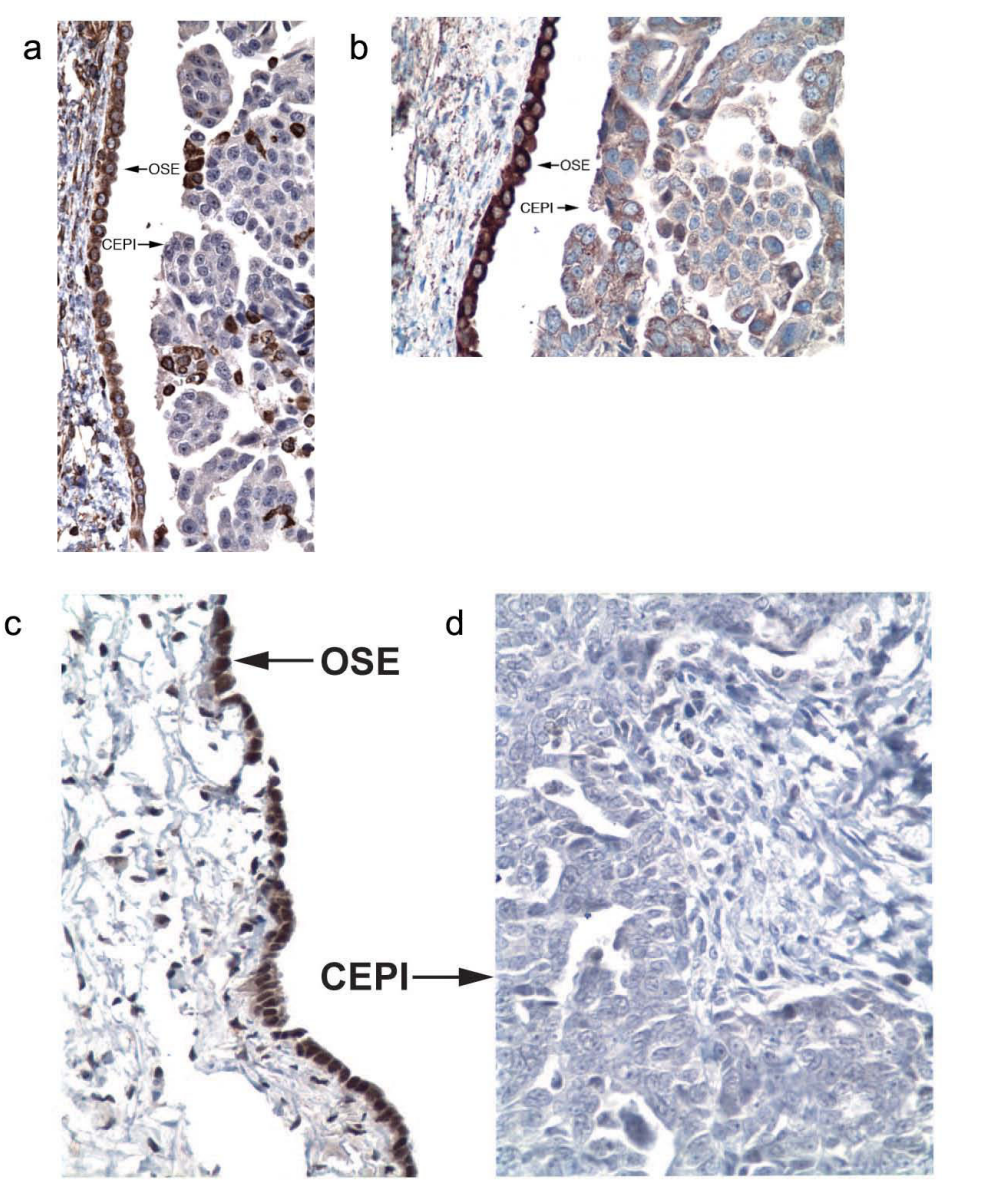
**Immunohistochemistry of OSE gene products**. Immunohistochemistry was performed on fresh frozen paraffin embedded OSE and CEPI tissue samples. Staining with primary antibodies against (A) secreted frizzled-related protein 1 (SFRP1), (B) aldehyde dehydrogenase 1 family, member A2 (ALDH1A2), and (C and D) lim homeobox 9 (LXH9) indicated strong protein expression in OSE (labeled arrow) and lower or absent protein expression in CEPI (labeled arrow), consistent with mRNA expression values shown in Additional file 5. Each image is a typical representative from 10 normal and 10 cancer slides. The slides chosen for display contain both CEPI and normal adjacent OSE from the same tissue sample.

Figure 4 affirms the corresponding protein expression levels for a number of the differentially expressed genes identified by microarray analysis. Immunohistochemistry was performed on formalin fixed, paraffin-embedded (FFPE) archived tissue samples selected to contain both ovarian serous adenocarcinoma and adjacent OSE. Consistent with the microarray profiles, we found that SFRP1 (Figure 4a), ALDH1A2 (Figure 4b), and LHX9 (Figure 4c and 4d) are differentially expressed at the protein level between OSE and CEPI. We are currently performing immunohistochemistry for additional proteins in order to substantiate the activity of the pathways discussed above.

### Gene set enrichment analyses identify overlapping gene expression signatures of specific cell functions in OSE and CEPI

In order to provide a comparative gene expression perspective to the differentially expressed genes identified in our study, we employed Gene Set Enrichment Analysis (GSEA) to compare our results to a large collection of previously curated biological experiments (e.g., expression microarray, predicted *cis*-regulatory motifs, etc.) [58]. The Molecular Signatures Database (MSigDB) is the collection of gene sets for use with GSEA software [58]. Statistically significant overlaps between differentially expressed gene sets derived from independent expression microarrays provide evidence for shared biological functions between the interrogated experiments. GSEA allows for the ranked comparison of differentially expressed genes between independent experiments. Genes from our dataset were ranked by their average fold difference in expression between OSE to CEPI. GSEA was then used to identify gene sets from previously published studies that significantly overlap with genes differentially expressed between CEPI and OSE.

#### Similarities between OSE and cancer stem cells

The gene set with the highest GSEA normalized enrichment score (NES, -2.58, p = 3.31 e^-23^) to OSE was BOQUEST_CD31PLUS_VS_CD31 MINUS_DN (see Additional file 7). This gene set was derived from previously reported differences between the transcriptional signatures of stem cell-like (CD31^-^) and differentiated (CD31^+^) adipose tissue cells [59].

The cancer stem cell model posits that only a specific subset of a cancer cell population is able to sustain tumor growth [60]. Putative ovarian cancer stem cells (OCSC) have recently been isolated from disaggregated ovarian serous adenocarcinomas and from ascites fluid [61,62]. The origin of OCSCs has yet to be determined but they were shown to be CD44^+ ^(Indian blood group), and KIT^+ ^(Zuckerman 4 feline sarcoma viral oncogene homolog) [61,62]. While our results indicate that *CD44 *is highly expressed in OSE and expressed at significantly lower levels in CEPI, *KIT *expression was found to be low in all of our samples and thus not differentially expressed. However, the default Affymetrix MAS5 absent/present calls detected the presence of *KIT *in 5 of our 12 OSE samples but absent in all 12 CEPI samples suggesting that KIT is expressed in OSE. In addition, we found that KIT ligand (*KITLG*), is highly expressed in OSE and significantly lower expressed in CEPI. These findings indicate that OCSC and OSE express a number of overlapping genes and support the notion that OSE could give rise to OCSC.

The capacity for self-renewal in multiple adult stem cell lineages has been associated with expression of the LIM homeobox genes [63,64]. Our results demonstrate that LIM homeobox 1, 2, 6 and 9 (*LHX1*, *LHX2*, *LHX6 *and *LHX9*) are differentially expressed between OSE and CEPI. *LHX2 *and *9 *are highly expressed in OSE and not expressed in CEPI, whereas *LHX1 *and *LHX6 *are expressed in CEPI and not in OSE.

#### Similarities between wound healing and CEPI

The gene set with the highest positive GSEA normalized enrichment score (NES, 3.04, p = 5.9 e^-64^) to CEPI was SERUM_ FIBROBLAST_ CELLCYLE (see Additional file 8). This set was derived from a previously reported overlap between transcriptional signatures in common between wound healing and poor cancer prognosis [65]. Many of the genes from this set that are highly expressed in CEPI promote entry into and progression through the cell cycle (e.g., *FOXM1*, *PTTG1*, *AURKA*) and have been previously associated with stage III epithelial ovarian cancers [66,67].

## Discussion

We have found that over 2000 genes are significantly differentially expressed between OSE and CEPI. Many of these genes are known to be involved in the canonical cell cycle pathway, as well as, signaling pathways previously implicated in development (i.e., the TGFB/BMP, WNT, NOTCH, Hedgehog and Retinoid pathways). The fact that many of the genes highly expressed in OSE have previously been associated with the maintenance of stem cells in a quiescent state is relevant to hypotheses on the cellular origin of ovarian cancer.

Under the dedifferentiation hypothesis of cancer development, cancer cells are postulated to be less differentiated than their progenitor cells but often resemble their tissue of origin phenotypically [5]. The fact that CEPI appear more rather than less differentiated than OSE and do not phenotypically resemble OSE has been offered as evidence that these cells are not the source of CEPI [7]. An alternative hypothesis is that OSE are stem-cell like and maintain a degree of pluripotency sufficient to allow them to morphologically transform during the process of CEPI development. Our results indicate that many, if not all, of the OSE cells on the surface of the ovary are not terminally differentiated but arrested in a quiescent state characteristic of most adult stem cell populations [68]. While our findings are consistent with the hypothesis that OSE may retain properties of relatively uncommitted pluripotent cells until undergoing neoplastic transformation, they do not preclude the possibility that at least some tumors currently classified as ovarian may arise from related, yet anatomically distinct sources such as cancer initiating cells embedded within the fallopian tube [69]. Indeed, our finding that the OSE molecular profile so closely resembles that of previously identified somatic stem cells and cancer stem cells suggests that all ovarian cancer initiating cells, regardless of their proposed tissue of origin, will likely share many essential characteristics.

The processes by which stem cells self-renew and differentiate are accomplished by a combination of cellular division strategies known as symmetric and asymmetric division [17,70]. Symmetric division gives rise to two identical daughter stem cells. In contrast, asymmetric division results in one stem cell and one progenitor cell with limited self-renewal potential. Our results, showing that *LHX2 *and LHX9 are expressed in OSE supports the notion that asymmetric cellular division is occurring in OSE. Progenitor cells can subsequently go through several rounds of cell division before terminating into a mature differentiated cell. Whether or not stem cells self-renew or differentiate is regulated by the microenvironment. A microenvironment that is conducive to stem cell self-renewal is referred to as a stem cell niche [16,68]. Stem cell progeny that remain in a stem cell niche typically display arrested cell growth/replication and are described as being quiescent [68,71]. In contrast, stem cell progeny that exit a stem cell niche typically enter a transient period of accelerated cell division resulting in large numbers of cells prior to terminal differentiation.

The above description of stem cells and the niches that control their division is relevant to OSE because during the period between ovulations, OSE are quiescent. While arrested cell growth and division are associated with terminal differentiation, at least some OSE must not terminally differentiate because they reactivate their cell cycle and proliferate in response to ovulation. Evidence recently has been presented showing that cells on the surface of the macaque ovary transition from quiescent to a replicating phenotype in response to ovulation [72]. Similar phenomena have been previously observed in mice [73-75] and generally support the notion that ovulation temporarily disrupts the ovarian surface niche resulting in controlled proliferation.

Our results are consistent with the hypothesis that the surface of the ovary is an *interovulatory *or *facultative *stem cell niche and this suggests that all or many of the resting cells on the surface of the ovary are not terminally differentiated but arrested in a quiescent state characteristic of adult stem cell populations [68,71]. We find that a variety of signaling molecules (including TGFB/BMP and TGFBR family members, antagonists of the WNT and hedgehog signaling pathways, as well as members of the retinoid signaling pathway) is expressed at high levels in OSE. The fact that these molecules previously have been shown to be integral for the maintenance of stem cell niches in other organ systems [31,35,71,76,77] indicates that they are likely performing a similar function on the surface of the ovary between ovulations. Also consistent with the hypothesis that the surface of the ovary is a type of *facultative *stem cell niche is our finding that transcription factors previously implicated in self-renewal and asymmetric division are expressed in OSE [64,78]. In contrast, genes expressed in CEPI have been associated with the entry and progression of cells through the cell cycle [79-84]. Of particular note, is the high expression of the Cyclin E family genes (*CCNE*) in CEPI. It has recently been shown that the constitutive expression of CCNE in mouse embryonic stem cells results in an almost nonexistent G0/G1 phase of the cell cycle [68]. The lack of an extended G0/G1 results in less time for cells to respond to mitogens that stimulate cellular differentiation. Thus, the elevated expression of *CCNE *in CEPI may contribute to cancer growth in a similar fashion.

The complex molecular processes underlying the onset and development of epithelial ovarian cancer is only beginning to be unraveled. Our results indicate that a number of key developmental pathways are involved in the establishment and development of ovarian cancer. While many of these pathways have previously been either directly or indirectly implicated in ovarian cancer, detailed network analyses of our gene expression data led to the identification of linkages between these pathways attributable to the altered expression of key regulatory genes. We believe that the type of detailed network analyses of gene expression data presented in this paper when combined with next generation sequencing for mutation analyses of individual ovarian adenocarcinoma genomes will help expand our understanding of the origins of ovarian cancer and facilitate the development of more effective therapies.

## Conclusion

Accumulating evidence suggests that somatic stem cells undergo mutagenic transformation into cancer initiating cells. Our results indicate that OSE express many genes involved in somatic stem cell maintenance. These findings are consistent with the hypothesis that human OSE retain properties of relatively uncommitted multipotent progenitor cells until undergoing neoplastic transformation. The multipotent nature of OSE may contribute to the complex histological subtypes of epithelial ovarian cancer (e.g., serous, endometrioid, clear cell, mucinous and others). While our findings do not rule out the possibility that ovarian cancers may also arise from other sources, they are *inconsistent *with claims that ovarian surface epithelia cannot serve as the origin of ovarian cancer initiating cells.

## Competing interests

The authors declare that they have no competing interests.

## Authors' contributions

LW and LM carried out the microarray experiments. SL performed and interpreted the immunohistochemistry. BB and KT aided in the coordination of the study and the collection of samples for study. NB participated in the design of the study, the analysis and interpretation of the microarray data and drafted the manuscript. JM conceived of the study, participated in its design and coordination and drafted the manuscript. All authors read and approved the final manuscript.

## Pre-publication history

The pre-publication history for this paper can be accessed here:

http://www.biomedcentral.com/1755-8794/2/71/prepub

## Supplementary Material

Additional file 1 **supplemental_table_1_2915.xls**. differentially expressed Affymetrix probe sets. Click here for file

Additional file 2 **supplemental_table_2_2320.xls**. differentially expressed genes. Click here for file

Additional file 3 **supplemental_table_3_oci_expo_mda_intersections.xls**. OCI, EXPO, and MD Anderson data intersection probe set lists. Click here for file

Additional file 4 **supplemental_table_4_go_p_05_genomica.xls**. Gene Ontology List. Click here for file

Additional file 5 **supplemental_figure_1.tif**. heat map of genes in figure 3. Click here for file

Additional file 6 **supplemental_figure_1_legend.doc**. supplemental_figure_1_legend. Click here for file

Additional file 7 **supplemetal_table_5_63_overlap_genes.xls**. 63 Gene Overlap between OSE gene set and BOQUEST_CD31PLUS_VS_CD31 MINUS_DN gene set from MSigDB. Click here for file

Additional file 8 **supplemetal_table_6_65_overlap_genes.xls**. 65 Gene Overlap between CEPI gene set and SERUM_FIBROBLAST_CELLCYCLE gene set from MSigDB. Click here for file
