# Supplementary figures and images for: Gene expression profiling supports the hypothesis that human ovarian surface epithelia are multipotent and capable of serving as ovarian cancer initiating cells
Source: BMC Med Genomics. 2009 Dec 29;2:71. doi: 10.1186/1755-8794-2-71 (PMC2806370; doi:10.1186/1755-8794-2-71)

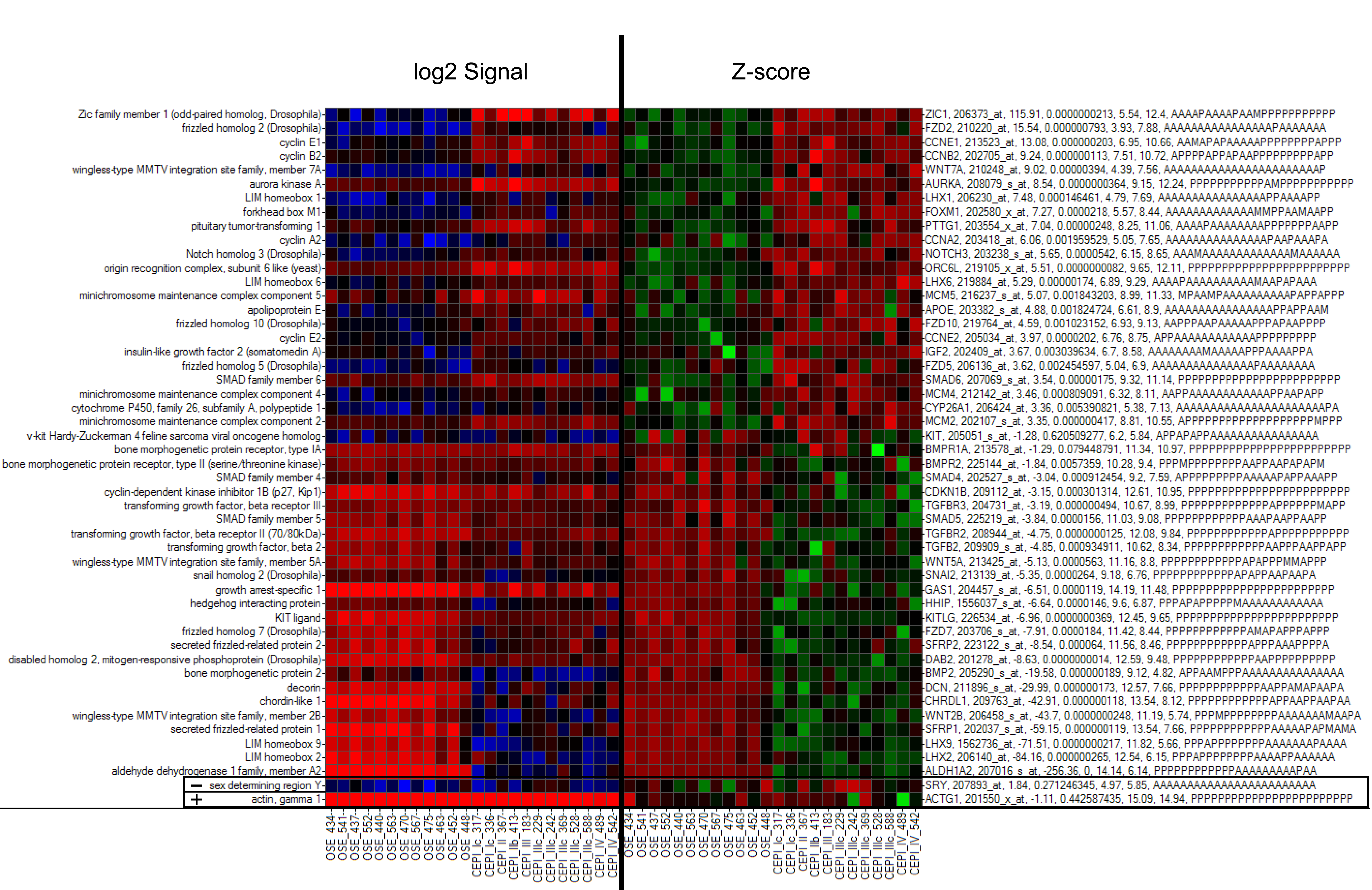

Supplement: Additional file 1 — supplemental_table_1_2915.xls. differentially expressed Affymetrix probe sets. [file 1755-8794-2-71-S1.TIFF]
