# Supplementary material for: Gene expression profiling supports the hypothesis that human ovarian surface epithelia are multipotent and capable of serving as ovarian cancer initiating cells
Source: BMC Med Genomics. 2009 Dec 29;2:71. doi: 10.1186/1755-8794-2-71 (PMC2806370; doi:10.1186/1755-8794-2-71)
Supplement: Additional file 8 — supplemetal_table_6_65_overlap_genes.xls. 65 Gene Overlap between CEPI gene set and SERUM_FIBROBLAST_CELLCYCLE gene set from MSigDB. [file 1755-8794-2-71-S8.DOC]

**Supplemental Figure 1. Log2 signal and Z-score heat maps of individual genes discussed in text.**

The signal heat map on the left indicates the log2 signal for each gene colored from low (blue) to high (red). The blue to red transition point (black) was chosen as 7, which equals the log2 of a signal intensity value of 2^7^ or 128. The Z-score heat map is on the right as described in Figure 1. The genes are labeled on the left by gene title and on the right by gene symbol, fold change from NOSE to CEPI, t-test p value, NOSE average log2, CEPI average log2, and Affymetrix “absent” (A) and “present” (P) calls. Samples are labeled at the bottom of each column. Actin, gamma 1 (*ACTG1*) and sex determining region Y (*SRY*) were chosen as positive and negative signal controls, respectively.
